# Impact of Early C-Reactive Protein/Albumin Ratio on Intra-Hospital Mortality Among Patients with Spontaneous Intracerebral Hemorrhage

**DOI:** 10.3390/jcm9041236

**Published:** 2020-04-24

**Authors:** Michael Bender, Kristin Haferkorn, Michaela Friedrich, Eberhard Uhl, Marco Stein

**Affiliations:** Department of Neurosurgery, Justus-Liebig-University Gießen, Klinikstraße 33, 35392 Gießen, Germany; kristin.haferkorn@uk-gm.de (K.H.); michaela.friedrich@neuro.med.uni-giessen.de (M.F.); eberhard.uhl@neuro.med.uni-giessen.de (E.U.); marco.stein@neuro.med.uni-giessen.de (M.S.)

**Keywords:** C-reactive protein/albumin ratio, Intensive care unit treatment, Intracerebral hemorrhage, Intra-hospital mortality

## Abstract

Objective: The impact of increased C-reactive protein (CRP)/albumin ratio on intra-hospital mortality has been investigated among patients admitted to general intensive care units (ICU). However, it was not investigated among patients with spontaneous intracerebral hemorrhage (ICH). This study aimed to investigate the impact of CRP/albumin ratio on intra-hospital mortality in patients with ICH. Patients and Methods: This retrospective study was conducted on 379 ICH patients admitted between 02/2008 and 12/2017. Blood samples were drawn upon admission and the patients’ demographic, medical, and radiological data were collected. The identification of the independent prognostic factors for intra-hospital mortality was calculated using binary logistic regression and COX regression analysis. Results: Multivariate regression analysis shows that higher CRP/albumin ratio (odds ratio (OR) = 1.66, 95% confidence interval (CI) = 1.193–2.317, *p* = 0.003) upon admission is an independent predictor of intra-hospital mortality. Multivariate Cox regression analysis indicated that an increase of 1 in the CRP/albumin ratio was associated with a 15.3% increase in the risk of intra-hospital mortality (hazard ratio = 1.153, 95% CI = 1.005–1.322, *p* = 0.42). Furthermore, a CRP/albumin ratio cut-off value greater than 1.22 was associated with increased intra-hospital mortality (Youden’s Index = 0.19, sensitivity = 28.8, specificity = 89.9, *p* = 0.007). Conclusions: A CRP/albumin ratio greater than 1.22 upon admission was significantly associated with intra-hospital mortality in the ICH patients.

## 1. Introduction

Prediction of intra-hospital mortality in patients with spontaneous intracerebral hemorrhage (ICH) is still a challenging measure for health professionals. The well-known prognostic factors predicting poor outcomes after ICH include advanced age, lower level of consciousness, lower Glasgow Coma Scale (GCS) score upon admission, larger volume and expansion of ICH, lager peri-hemorrhagic edema, and presence of hydrocephalus and intraventricular hemorrhage (IVH) [1,2,3,4,5,6]. In spite of advancement in intensive care medicine, ICH patients still have a high 30-day mortality (up to 52%) or survive with significant neurological disabilities [7,8,9,10,11,12]. Neurological deterioration due to expansion of ICH occurs in up to 30% of the patients within the first 24 hours. Therefore, this period of ICU treatment is essential for the determination of patient prognosis.

Serum biomarkers could be helpful to make early decisions concerning the future directions for ICU treatments. Elevated values of white blood cell count, troponin I, cortisol, blood glucose level, and C-reactive protein (CRP) are associated with poor outcome after ICH [10,13,14,15,16,17]. However, the clinical benefits of these biomarkers are controversial [10,13,14,15,16,17]. An elevated CRP/albumin ratio upon admission is a well-known and commonly used independent predictor of mortality among the patients admitted to the general intensive care units (ICUs) [18,19,20]. CRP, as an acute-phase protein, followed a cytokine-induced stimulation as a result of trauma, ischemia, or inflammation [20,21]. Lobo et al. reported an increased risk of mortality and organ failure in patients with elevated CRP upon ICU admission [22]. Furthermore, hypoalbuminemia, as an indicator of the current nutritional status and liver synthesis function, occurs frequently in critically ill patients and is associated with increased mortality [23]. Moreover, increased CRP value is commonly evidenced in patients with cancer or infections, although decreasing albumin values occurs frequently during serious infections in otherwise healthy patients. However, the association between CRP/albumin ratio upon admission and intra-hospital mortality is still unknown among the patients with ICH admitted to neurosurgical ICUs. Early identification of ICH patients with a high risk for intra-hospital mortality could be useful to estimate the patient prognosis and determine the future directions for ICU treatments. With this background in mind, the current study was conducted to investigate the impact of CRP/albumin ratio, as an early serum biomarker, on intra-hospital mortality among neurosurgical ICU-admitted patients with spontaneous ICH.

## 2. Materials and Methods

### 2.1. Study Design and Population

This retrospective study was conducted on all spontaneous ICH patients who were admitted to the ICU, of the Neurosurgical Department of the University Hospital Gießen, between February 2008 and December 2017 and treated for at least 24 h. The diagnosis of ICH was established by computed tomography (CT). The exclusion criteria were: 1) diagnosis of ICH due to trauma (*n* = 128), vascular malformation (*n* = 133), or neoplasia (*n* = 93), 2) present of acute and/or chronic liver failure (*n* = 26), and 3) age of < 18 years. The study protocol was approved by the Ethics Committee of the Justus-Liebig-University, Gießen, Germany (No. 95/17, February 19, 2019).

### 2.2. Data Collection

The baseline data, including comorbidities, premedication, treatment regimen, cardiopulmonary parameters (CP) within the first 24 h, serum biomarkers, radiological data, and intra-hospital outcome at discharge and mortality, duration of hospital stay, body mass index, acute physiology and chronic health evaluation II (APACHE II) score, and GCS score upon admission were extracted from the electronic medical records of the patients [24,25].

Comorbidities were analyzed regarding the presence of chronic arterial hypertension, chronic obstructive pulmonary diseases, cardiac arrhythmia, coronary artery diseases, heart failure, history of cardiac/cardiosurgical intervention, chronic renal insufficiency, diabetes mellitus, cancer, and history of ischemic stroke or ICH. In addition, evaluation of premedication included absence of premedication, antihypertensive, anti-obstructive, antidiabetic, and antiplatelet agents, as well as new oral anticoagulants, vitamin K antagonists.

### 2.3. Treatment Regimen and Intensive Care Unit Treatment

After the initial examination in the emergency room and confirmation of ICH diagnosis, all patients were treated either immediately or after emergency surgery at the neurosurgical ICU for at least 24 h. Cardiopulmonary monitoring was routinely performed with an invasive blood pressure measurement catheter (Combitrans Monitoring Set arteriell; B. Braun, Melsungen, Germany), a pulse oximeter (Nellcor adult SpO2 sensor; Covidien LLC, Mansfield, MA, USA), and a 3-lead electrocardiogram (B. Braun, Melsungen, Germany). Furthermore, systolic arterial blood pressure was kept between 120 and 140 mmHg during the first 14 days. The target of arterial oxygen partial pressure was maintained at ≥ 100 mmHg. In addition, blood gas analysis (ABL800 FLEX; Radiometer, Copenhagen Denmark, and Krefeld, Germany) was performed based on a four-hour interval by taking arterial blood samples. Additionally, all patients received central venous catheters (Arrow International, Inc., Reading, PA, USA) for the administration of intravenous drugs. In case of respiratory insufficiency or a GCS score of less than 9, endotracheal intubation and mechanical ventilation were begun with a pressure-controlled mode (Servo-I; Maquet, Rastatt, Germany). Continuous analgosedation was performed using midazolam (5–40 mg/h) or propofol (200–500 mg/h), along with sufentanil (35–100 µg/h). Furthermore, all ICH patients who were admitted to our neurosurgical department and were on anticoagulation and/or antiplatelets received reversal agents (prothrombin agents) and/or platelet transfusion, regardless if a surgical or medical treatment was carried out. Specific factor antidotes for new oral anticoagulants were not used during the study period. The indication for medical or additional surgical treatment was made by the neurosurgeon consultant in accordance with clinical (e.g., level of consciousness or presence of neurological deficit) and/or radiological (e.g., growth of ICH or midline shift) conditions of the patients. The medical treatments included all modalities of conservative ICU treatments. Additional surgical treatments consisted of the following: the insertion of an external ventricular drain, the evacuation of ICH, decompressive craniectomy or decompressive craniectomy with evacuation of ICH. The medical records of all patients were analyzed in terms of the performed treatment regimen.

### 2.4. Cardiopulmonary Parameters

The CP included median systolic blood pressure, heart rate, positive end-expiratory pressure level, arterial oxygen partial pressure, intubation status and body temperature upon admission as well as average norepinephrine application rate (NAR) and average inspiratory oxygen fraction (FiO_2_) during the first 24 h of the ICU treatment. These data were recorded continuously within five-minute intervals and stored in the digital ICU data recording system.

### 2.5. Serum Biomarkers 

Blood samples were drawn immediately after the patients’ admission to the emergency department. The serum biomarkers evaluated upon admission included white blood cell count in giga/L (XE 5000 Hematology Analyzer; flow cytometric, Sysmex, Germany), hemoglobin value in g/dl (XE 5000 Hematology Analyzer; photometric, Sysmex, Germany), hematocrit value in % (XE 5000 Hematology Analyzer; cumulative pulse height summation, Sysmex, Germany), cholinesterase in U/L (ADVIA Chemistry XPT^®^ CHE Assay, Siemens, Germany), blood glucose level in mg/dl (ADVIA Chemistry XPT^®^ GLUH_c Assay, Siemens, Germany), serum lactate level in mmol/L (ADVIA Chemistry XPT^®^ LAC Assay, Siemens, Germany), troponin I in µg/dl (ADVIA Centaur XPT^®^, TNI-Ultra Assay, Siemens, Germany), cortisol value in µg/dl (ADVIA Centaur XPT^®^, Cortisol Assay, Siemens, Germany), CRP in mg/L (ADVIA Chemistry XPT^®^ wrCRP Assay, Siemens, Germany), and albumin level in g/L (ADVIA Chemistry XPT^®^ ALB_c Assay, Siemens, Germany). Moreover, the CRP/albumin ratio was calculated by the division of the CRP value to the albumin value obtained at the time of patient admission.

### 2.6. Radiological Data

The initial CT scan was analyzed regarding the localization (deep and lobar supratentorial vs. infratentorial) and the volume of the ICH. The formula A * B * C/2 was used to calculate the ICH volume. Furthermore, the presence of hydrocephalus (Evans’ Index > 0.3) and IVH (Graeb score > 1) was evaluated and analyzed [26,27]. All CT scans were analyzed by three independent individuals (i.e., M.S., K.H., and M.B.), without significant difference, in regard to ICH volume and presence of hydrocephalus and IVH, between the individuals.

### 2.7. Intra-Hospital Outcome and Mortality

Evaluation of intra-hospital outcome and mortality was performed using the Modified Rankin Scale (mRS) at discharge [28].

### 2.8. Statistical Analysis

The data were expressed as mean ± SD and median/interquartile range (IQR) for the normally and non-normally distributed parameters, respectively. The total study population was classified into survivors and non-survivors in terms of intra-hospital outcome. The Chi-square test and Student’s t-test were utilized to identify univariate differences between the two groups. The univariate analysis was performed in Prism software (Version 5; GraphPad Software, Inc., La Jolla, CA, USA). A p-value less than 0.05 was considered statistically significant.

After performing univariate analysis, all parameters that obtained the level of significance were further investigated using a multivariate binary logistic analysis with a forward stepwise method. The variables were removed based on the likelihood ratio and a Cox regression hazard model in relation to intra-hospital mortality. Afterward, the data were analyzed in SPSS software (Version 15.0; SPSS Inc., Chicago, USA), and cut-off value were calculated for the CRP/albumin ratio concerning the increased intra-hospital mortality. The Youden’s index and area under the curve were calculated in a receiver operating curve (ROC) analysis using R statistical software (Version 3.4.1, RCore Team 2017, Dormagen, Germany).

## 3. Results

### 3.1. Main Characteristics

**Figure 1:**
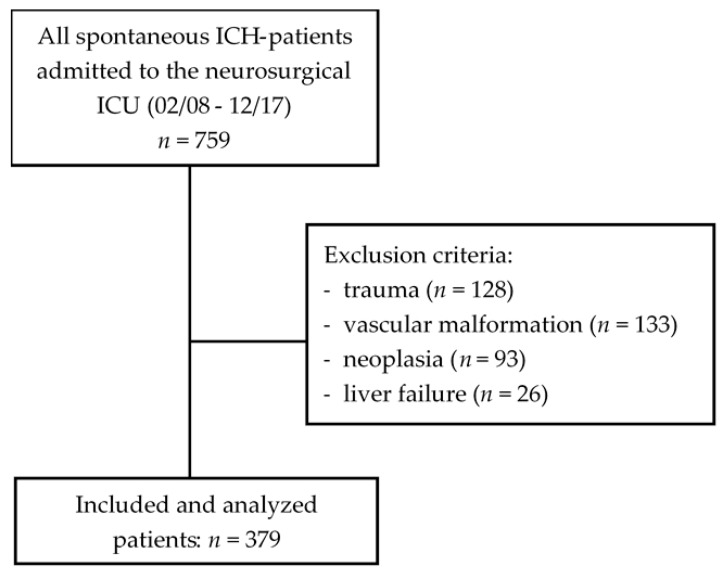
Flowchart for inclusion and exclusion criteria.

This study included 379 patients (Figure 1) with a mean age of 68.2 ± 13.3 years (age range: 18–93 years), out of whom 209 cases were male. A median GCS score of 8 (IQR: 3–12) and APACHE II score of 14 (IQR: 11–19) were recorded upon admission.

The most common comorbidity was chronic arterial hypertension (60.4%), followed by cardiac arrhythmia (19.3%). Moreover, antihypertensive drugs (46.4%) and vitamin K antagonists (20.1%) were the most frequent premedication. However, 46.2% of the patients had no history of premedication. A total of 226 (59.6%) patients were intubated and mechanically ventilated within the first 24 h. In addition, mean NAR (0.03 ± 0.04 µg/kg/min) and FiO_2_ (34.8 ± 13.4) values were required to achieve the cardiopulmonary targets within the first 24 h of the ICU treatment. The mean CRP and albumin values upon admission were 22.1 ± 38.9 mg/L and 38.1 ± 5.6 g/L, respectively, the mean CRP/albumin ratio was 0.63 ± 1.1.

163 (43%) patients were treated conservatively and 216 (57%) patients required additional surgical treatment. The most common surgical procedure was the insertion of EVD (38.4%). The mean ICH volume was 51.8 ± 42.3 cm^3^ (range: 1.0–223.4 cm^3^), and deep supratentorial was the most frequent localization of ICH. Furthermore, the presence of hydrocephalus was confirmed in 42.7% of the patients, and 71% of them suffered from IVH. Table 1 summarizes the main characteristics of all patients.

### 3.2. Intra-Hospital Mortality and Outcome

The median mRS at discharge was 5 (range: 4–6) and 31.1% died within inpatient treatment. A median GCS score of 4 (IQR: 3–7) and APACHE II score of 20 (IQR: 18–22.3) was found in the cohort of non-survivors, which differ significantly from the cohort of survivor with a GCS of 10 (IQR: 6–13; *p* < 0.0001) and an APACHE II score of 13 (IQR: 10–15; *p* < 0.0001). Furthermore, intra-hospital mortality was significantly associated with advanced age (*p* < 0.0001), preexisting heart failure (*p* = 0.0003), absence of known chronic arterial hypertension (*p* = 0.01), absence of premedication (*p* < 0.0001), and consumption of antihypertensive medication (*p* = 0.0004). The non-survivor group required significant more NAR (0.03 ± 0.02 µg/kg/min)) and FiO_2_ (0.37 ± 0.12) in comparison to the cohort of survivors, with an mean NAR of 0.02 ± 0.04 µg/kg/min (*p* = 0.01) and FiO_2_ of 0.34 ± 0.14 (*p* = 0.004), within the first 24 h of the ICU treatment to accomplish the cardiopulmonary targets. In addition, intra-hospital mortality significantly correlated with a higher rate of intubation (*p* < 0.0001), lower body temperature (*p* < 0.0001), lower cholinesterase level (*p* = 0.0003), lower albumin level (*p* = 0.0002), higher blood glucose level (*p* = 0.003), higher CRP level (*p* = 0.02), higher CRP/albumin ratio (*p* = 0.01), infratentorial localization of ICH (*p* = 0.01), presence of IVH (*p* = 0.0002), and hydrocephalus (*p* = 0.001; Table 2). Furthermore, the cohort of non-survivors had a significant lager mean ICH volume (72.9 ± 49.9 cm^3^) compared to the cohort of survivors (42.3 ± 34.5 cm^3^; *p* < 0.0001).

**Figure 2:**
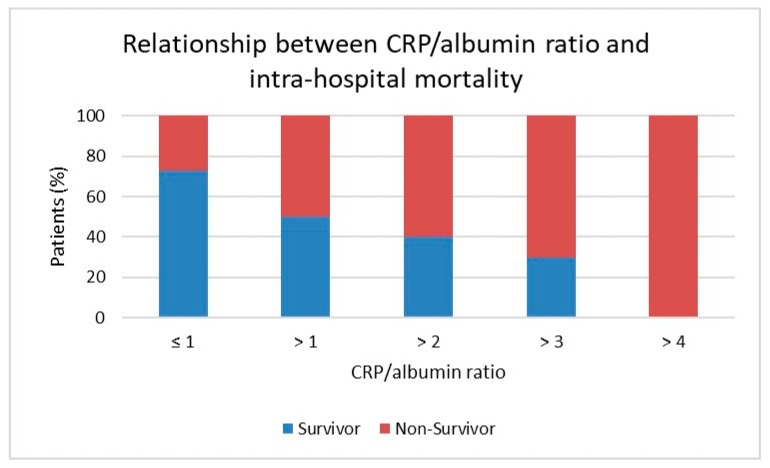
Relationship between C-reactive protein (CRP)/albumin ratio and intra-hospital mortality.

According to the results of the multivariate binary logistic analysis, the independent predictors of intra-hospital mortality upon admission were advanced age (odds ratio (OR) = 1.06, 95% confidence interval (CI) = 1.031–1.096, *p* < 0.0001), lower GCS score (OR = 0.83, 95% CI = 0.752–0.918, *p* < 0.0001), higher APACHE II score (OR = 1.33, CI = 1.223–1.454, *p* < 0.0001), preexisting heart failure (*p* = 0.019, OR = 4.05, CI = 1.254–13.06), absence of premedication (OR = 2.34, 95% CI = 1.034–5.29, *p* = 0.041), larger volume of intracerebral hematoma (OR = 1.016, 95% CI = 1.008–1.025, *p* < 0.0001), and higher CRP/albumin ratio (OR = 1.66, 95% CI = 1.193–2.317, *p* = 0.003). Furthermore, ROC analysis indicated a significant association between intra-hospital mortality and a CRP/albumin ratio cut-off value of > 1.22 (Youden’s Index 0.19, sensitivity: 28.8, specificity: 89.9, *p* = 0.007) upon admission. Table 3 presents the results of the multivariate Cox regression hazard analysis. As the results indicated, an increase of 1 in the CRP/albumin ratio was associated with a 15.3% increase in the risk of intra-hospital mortality (hazard ratio (HR) = 1.153, 95% CI = 1.005–1.322, *p* = 0.42). Furthermore, the risk of intra-hospital mortality increased with higher CRP/albumin ratio, as shown in Figure 2.

## 4. Discussion

### 4.1. Summary of Findings

This retrospective study was conducted on 379 patients to assess the impact of CRP/albumin ratio on intra-hospital mortality among neurosurgical ICU patients with ICH. Similar to previous studies, the independent predictors of intra-hospital mortality in ICH patients were found to be advanced age, lower GCS score, higher APACHE II score, and larger volume of intracerebral hematoma after admission [6,7,8,11,12,20]. However, in the current study, a higher CRP/albumin ratio upon admission was identified as a new independent and additional predictor of intra-hospital mortality among the patients. The impact of CRP/albumin ratio on intra-hospital mortality has been frequently investigated in patients admitted to general ICUs but not in patients with ICH admitted to neurosurgical ICUs [18,19,20,29]. According to our results, a CRP/albumin ratio greater than 1.22 upon admission was significantly associated with intra-hospital mortality. Furthermore, an increase of 1 in the CRP/albumin ratio was associated with a 15.3% increase in the risk of intra-hospital mortality among the patients. Therefore, the value of CRP/albumin ratio could be helpful to estimate the prognosis and to determine the direction of further interventions in patients with ICH.

### 4.2. Intra-Hospital Mortality

In the current study, the overall intra-hospital mortality rate was 31.1%, which is consistent with the results obtained in the previous studies [6,7,8,11,12]. As in other studies, the intra-hospital mortality was significantly associated with advanced age, a lower initial GCS score, chronic arterial hypertension, preexisting heart failure, antihypertensive medication, a lower cholinesterase level, a higher blood glucose level, a larger ICH volume, a lower body temperature, a higher rate of intubated patients, infratentorial localization of ICH, and presence of IVH and hydrocephalus upon admission [1,2,3,4,5,6,9,10,11,13,15,16,17,21]. Moreover, patients with absence of premedication had increased intra-hospital mortality. This could be caused by unknown comorbidities due to no consulting of health care professionals, especially in patients with a less socioeconomic status as well as medical noncompliance or denial of risk factors, e.g., arterial hypertension, cardiac arrhythmia or diabetes mellitus, which could lead to absence of premedication. According to the results of the univariate regression analysis, higher values of NAR and FiO_2_ within the first 24 h of the ICU treatment were significantly associated with intra-hospital mortality. However, the results of the multivariate regression analysis showed no such correlation. Nevertheless, this emphasizes the importance of cardiopulmonary monitoring and ICU treatment within the first 24 h after admission to improve clinical outcomes following the incidence of ICH.

### 4.3. C-Reactive Protein/Albumin Ratio

CRP is an easily available positive acute-phase protein, which increases as a result of infection, ischemia, and/or trauma [20,21,30]. Therefore, CRP has been used as a prognostic factor in patients with heart failure, ICH, ischemic stroke, and sepsis [17,21,31,32]. Di Napoli et al. shows that an initially CRP elevation is an independent predictor for a higher risk of death in ICH patients [13]. Moreover, several studies reported on the independent negative influence of elevated CRP value upon admission on mortality and outcome in ICH patients [33,34]. In contrast, albumin is a negative acute-phase protein, which has been extensively investigated in the prediction of inflammatory diseases [20,33]. Patients with cancer or infections present commonly increased CRP values, but not decrease albumin value. Hypoalbuminemia occurred mostly during severe infections. Artero et al. reported hypoalbuminemia to be the most important prognostic factor in regard to mortality for community-acquired bloodstream infections with severe sepsis and septic shock [35]. However, the prognostic value of CRP and albumin in patients with reduced liver synthesis (e.g., malnutrition, liver cirrhosis, cancer, advanced age, or chronic heart failure) is limited. Therefore, the CRP/albumin ratio was established to combine information about the relation of systemic inflammation, dystrophia, and nutritional status in a new biomarker [20,21]. The CRP/albumin ratio has been already identified as a prognostic biomarker to assess outcomes in patients with sepsis, cancer, and chronic inflammatory diseases [18,21,30]. Moreover, several studies have extensively investigated the association between increased CRP/albumin ratio upon admission and mortality in patients admitted to the general ICUs [18,19,30].

In the present study, intra-hospital mortality correlated significantly with lower albumin value, as well as increased CRP and CRP/albumin values upon admission. This study revealed an increase in the CRP/albumin ratio upon admission as an independent predictor of intra-hospital mortality among ICH patients admitted to the neurosurgical ICU. The cut-off value of the CRP/albumin ratio was 1.22. An increase of 1 in the CRP/albumin ration was associated with a 15.3% increase in the risk of intra-hospital mortality.

Several studies have already defined cut-off values concerning patient outcomes; however, these values are highly variable [18,19,21,29,30]. Ranzani et al. suggested a cut-off value of 8.7 for the CRP/albumin ratio as a predictor of 90-day mortality in septic patients admitted to the ICU [18]. In contrast, Bai et al. proposed a cut-off value of 0.58 predicting 30-day unfavorable outcomes in neurocritically ill patients [21]. This discrepancy can be attributed to variations in the study populations. Inpatient admission to the general ICUs is frequently justified due to an infection (e.g., pneumonia or sepsis), acute heart decompensation (e.g., heart attack or unstable coronary artery diseases), or surgery (e.g., oncological surgery). These patients have higher inflammatory markers upon admission and hence a higher CRP/albumin ratio, as compared to neurocritically ill patients, in which the loss of consciousness is the main indication for ICU treatment.

The ICH score by Hemphill et al. is currently the most important clinical grading scale for early risk stratification in ICH patients concerning 30-day mortality [36]. The results of the current study suggest that the CRP/albumin ratio could be an additional early parameter to optimize the ICH score. However, this should be investigated in a further prospective study, especially to evaluate the correlation of the CRP/albumin ratio and the results of the ICH score concerning mortality in ICH patients.

Nevertheless, our findings suggested that CRP/albumin ratio upon admission predicted intra-hospital mortality among the patients with ICH admitted to the neurosurgical ICUs. Therefore, CRP/albumin ratio can be considered a helpful early biomarker to not only early identification of patients with a high risk of intra-hospital mortality but also direct further interventions.

### 4.4. Limitations and Strengths of the Study

Although this study includes some strengths, it suffers from several limitations. Therefore, the findings should be interpreted with caution. First of all, because of the retrospective design of the study, we have no repeated measurements of CRP and albumin values and therefore CRP/albumin ratios. Secondary, our findings suggest that the CRP/albumin ratio is prognostic for intra-hospital mortality, however significantly lower ICH volumes, lower APACHE II scores and higher GCS scores were observed in the cohort of non-survivor compared to the survivor group. This limitation could be explained by the retrospective study design and should investigate in a further prospective study. Finally, the findings cannot be applied to all types of ICH due to the exclusion of patients with trauma, vascular malformations, or malignancy.

On the other hand, the key strength of the present study was the large study population with comprehensive demographic characteristics, as well as cardiopulmonary, radiological, and laboratory chemistry data records. Furthermore, to the authors’ knowledge, this study was the first report evaluating the association of CRP/albumin ratio and intra-hospital mortality of patients with ICH admitted to a neurosurgical ICU.

## 5. Conclusions

The CRP/albumin ratio seems to be an independent predictor of intra-hospital mortality among patients with ICH admitted to a neurosurgical ICU. Our data show a significant association between intra-hospital mortality and a CRP/albumin ratio greater than 1.22 upon admission. ICH patients with a CRP/albumin ratio ≤1.22 had a decreased risk of intra-hospital mortality. Therefore, this ratio could be one further important parameter of a score, with further well-known influencing parameters, to predict intra-hospital mortality.

## Figures and Tables

**Table 1:** Main characteristics of the study population (*n* = 379).

Parameter	Results
Baseline Data	
Age, Years, Mean (± SD) *	68.2 (13.3)
Women, *n* (%) *	170 (44.9)
Men, *n* (%) *	209 (55.1)
Body-Mass-Index, kg/m^2^, Median (IQR) *	26.2 (24.2–29.3)
Glasgow Coma Scale Score, Median (IQR) *	8 (3–12)
APACHE II Score, Median (IQR) *	14 (11–19)
Hospital Stay, Median (IQR) ***	16 (4–27)
Comorbidities	
Chronic Arterial Hypertension, *n* (%) *	229 (60.4)
Chronic Obstructive Pulmonary Diseases, *n* (%) *	16 (4.2)
Cardiac Arrhythmia, *n* (%) *	73 (19.3)
Coronary Artery Disease, *n* (%) *	48 (12.7)
Heart Failure, *n* (%) *	23 (6.1)
History of Cardiac/Cardiosurgical Intervention, *n* (%) *	42 (11.1)
Chronic Renal Insufficiency, *n* (%) *	22 (5.8)
Diabetes Mellitus, *n* (%) *	61 (16.1)
History of Ischemic Stroke, *n* (%) *	52 (13.7)
History of ICH, *n* (%) *	17 (4.5)
Cancer, *n* (%) *	30 (7.9)
Premedication	
Absence of Premedication, *n* (%) *	175 (46.2)
Antihypertensive Drugs, *n* (%) *	176 (46.4)
Antiobstructive Drugs, *n* (%) *	5 (1.3)
Antidiabetic Drugs, *n* (%) *	39 (10.3)
Antiplatelet Agents, *n* (%) *	53 (14.0)
New Oral Anticoagulants, *n* (%) *	13 (3.4)
Vitamin K Antagonist, *n* (%) *	76 (20.1)
Cardiopulmonary parameters	
Norepinephrine Application Rate, µg/kg/min, Mean (± SD) **	0.03 (0.04)
Systolic Blood Pressure, mmHg, Median (IQR) **	138 (129–146)
Heart Rate, Beats per Minute, Median (IQR) **	75 (64–87)
Inspiratory Oxygen Fraction, Mean (± SD) **	0.35 (0.13)
Intubated Patients, n (%) *	226 (59.6)
PEEP Level, Median (IQR) **	7 (6–9)
Arterial Oxygen Partial Pressure (mmHg), Median (IQR) **	109 (98–123)
Body Temperature, Centigrade, Median (IQR) *	36.3 (35.5–36.9)
Biomarkers	
White Blood Cells, giga/L, Mean (± SD) *	11 (4.6)
Hemoglobin, g/dL, Mean (± SD) *	13.1 (2.1)
Hematocrit, %, Mean (± SD) *	38.5 (5.6)
Cholinesterase, U/L, Mean (± SD) *	7805 (2264)
Blood Glucose, mg/dL, Mean (± SD) *	163.6 (59.2)
Serum Lactate, mmol/L, Mean (± SD) *	1.7 (1.5)
Troponin I, µg/dL, Mean (± SD) *	0.3 (2.6)
Cortisol, µg/dL, Mean (± SD) *	27.2 (18.9)
C-Reactive Protein, mg/L, Mean (± SD) *	22.1 (38.9)
Albumin, g/L, Mean (± SD) *	38.1 (5.6)
C-Reactive Protein/Albumin Ratio, Mean (± SD) *	0.63 (1.1)
Treatment	
Medical Treatment, *n* (%) ***	163 (43.0)
Additional Surgical Treatment, *n* (%) ***	216 (57.0)
Insertion EVD, *n* (%) ***	83 (38.4)
Evacuation ICH, *n* (%) ***	65 (30.1)
Decompressive Craniectomy, *n* (%) ***	19 (8.8)
Decompressive Craniectomy and Evacuation ICH, *n* (%) ***	49 (22.7)
Radiological Data	
Localization	
Supratentorial, Lobar, *n* (%) *	129 (34.0)
Supratentorial, Deep, *n* (%) *	194 (51.2)
Infratentorial, *n* (%) *	56 (14.8)
ICH Volume, cm^3^, Mean (± SD)	51.8 (42.3)
IVH, *n* (%) *	269 (71.0)
Hydrocephalus, *n* (%) *	162 (42.7)
Outcome	
mRS Score, Median (IQR) ****	5 (4–6)
mRS 0, *n* (%) ****	0 (0)
mRS 1, *n* (%) ****	21 (5.5)
mRS 2, *n* (%) ****	27 (7.1)
mRS 3, *n* (%) ****	26 (6.9)
mRS 4, *n* (%) ****	77 (20.3)
mRS 5, *n* (%) ****	110 (29.0)
mRS 6, *n* (%) ****	118 (31.1)

SD: standard deviation, IQR: interquartile range, APACHE II: Acute Physiology and Chronic Health Evaluation II, PEEP: positive end-expiratory pressure, EVD: external ventricular drain, ICH: intracerebral hemorrhage, IVH: intraventricular hemorrhage, mRS: modified Rankin Scale. * upon admission, ** within the first 24 hours, *** during inpatient treatment, **** at discharge.

**Table 2:** Univariate predictors of intra-hospital mortality.

Parameter	Survivor (*n* = 261)	Non-Survivor (*n* = 118)	*p*-Value
Baseline Data			
Age, Years, Mean (± SD) *	66.4 (13.5)	72.4 (11.8)	<0.0001
Women, *n* (%) *	125 (47.9)	45 (38.1)	0.07
Men, *n* (%) *	136 (52.1)	73 (61.9)	
Body-Mass-Index in kg/m^2^, Median (IQR) *	26.8 (24.2–29.4)	25.6 (23.9–27.8)	0.14
Glasgow Coma Scale Score, Median (IQR)*	10 (6–13)	4 (3–7)	<0.0001
APACHE II Score, Median (IQR) *	13 (10–15)	20 (18–22.3)	<0.0001
Hospital Stay, Median (IQR) ***	21 (13–32)	3 (1–8)	<0.0001
Comorbidities			
Chronic Arterial Hypertension, *n* (%) *	169 (64.8)	60 (50.8)	0.01
Chronic Obstructive Pulmonary Diseases, *n* (%) *	10 (3.8)	6 (5.1)	0.57
Cardiac Arrhythmia, *n* (%) *	46 (17.6)	27 (22.9)	0.23
Coronary Artery Disease, *n* (%) *	30 (11.5)	18 (15.3)	0.31
Heart Failure, *n* (%) *	11 (4.2)	12 (10.2)	0.0003
History of Cardiac/Cardiosurgical Intervention, *n* (%) *	25 (9.6)	17 (14.4)	0.17
Chronic Renal Insufficiency, *n* (%) *	13 (6.1)	9 (7.6)	0.31
Diabetes Mellitus, *n* (%) *	43 (16.5)	18 (15.3)	0.76
History of Ischemic Stroke, *n* (%) *	39 (14.9)	13 (11)	0.06
History of ICH, *n* (%) *	9 (3.5)	8 (6.8)	0.15
History of Cancer, *n* (%) *	19 (7.3)	11 (9.3)	0.5
Premedication			
Absence of Premedication, *n* (%) *	101 (38.7)	74 (62.7)	<0.0001
Antihypertensive Drugs, *n* (%) *	137 (52.5)	39 (33.1)	0.0004
Antiobstructive Drugs, *n* (%) *	2 (0.8)	3 (2.5)	0.16
Antidiabetic Drugs, *n* (%) *	25 (9.6)	14 (11.9)	0.5
Antiplatelet Agents, *n* (%) *	36 (13.8)	17 (14.4)	0.87
New Oral Anticoagulants, *n* (%) *	6 (2.3)	7 (5.9)	0.07
Vitamin K Antagonist, *n* (%) *	52 (19.9)	24 (20.3)	0.93
Cardiopulmonary Parameter			
Norepinephrine Application Rate, µg/kg/min, Mean (± SD) **	0.02 (0.04)	0.03 (0.02)	0.01
Systolic Blood Pressure, Median (IQR) **	139 (131–146)	136 (126–146)	0.13
Heart Rate, Beats per Minute, Median (IQR) **	75 (66–88.5)	74 (60.8–85)	0.07
Inspiratory Oxygen Fraction, Mean (± SD) **	0.34 (0.14)	0.37 (0.12)	0.004
Intubated Patients, n (%) *	138 (52.9)	88 (74.6)	<0.0001
PEEP Level, Median (IQR) **	7 (6–9)	8 (6–10)	0.7
Arterial Oxygen Partial Pressure (mmHg), Median (IQR) **	108 (98–123)	109 (99–125)	0.31
Body Temperature, Centigrade, Median (IQR) *	36.4 (35.8–37)	35.9 (35.1–36.5)	<0.0001
Biomarkers			
White Blood Cells, giga/L, Mean (± SD) *	10.7 (4.2)	11.7 (5.2)	0.26
Hemoglobin, g/dL, Mean (± SD) *	13.2 (2)	12.9 (2.2)	0.18
Hematocrit, %, Mean (± SD) *	38.8 (5.4)	38 (6.2)	0.24
Cholinesterase, U/L, Mean (± SD) *	8104 (2223)	7143 (2222)	0.0003
Blood Glucose, mg/dL, Mean (± SD) *	157.9 (54.8)	176.3 (66.6)	0.003
Serum Lactate, mmol/L, Mean (± SD) *	1.6 (1.3)	2 (1.7)	0.13
Troponin I, µg/dL, Mean (± SD) *	0.1 (0.5)	0.6 (4.1)	0.48
Cortisol, µg/dL, Mean (± SD) *	26.7 (19)	28.5 (18.8)	0.41
C-Reactive Protein, mg/L, Mean (± SD) *	18 (34.5)	31.4 (46.2)	0.02
Albumin, g/L, Mean (± SD) *	38.9 (5)	36.2 (6.3)	0.0002
C-Reactive Protein/Albumin Ratio, Mean (± SD) *	0.4 (0.9)	0.9 (1.5)	0.01
Treatment			
Medical Treatment, *n* (%) ***	106 (40.6)	57 (48.3)	0.16
Additional Surgical Treatment, *n* (%) ***	155 (59.4)	61 (51.7)	
Insertion EVD, *n* (%) ***	57 (36.8)	26 (42.6)	0.43
Evacuation ICH, *n* (%) ***	48 (31)	17 (27.9)	0.66
Decompressive Craniectomy, *n* (%) ***	13 (8.4)	6 (9.8)	0.74
Decompressive Craniectomy and Evacuation ICH, *n* (%) ***	37 (23.9)	12 (19.7)	0.51
Computed Tomography Scan			
ICH Volume, cm^3^, Mean (± SD) *	42.3 (34.5)	72.9 (49.9)	<0.0001
Localization of ICH			
Supratentorial, Lobar, *n* (%) *	84 (32.2)	45 (38.1)	0.26
Supratentorial, Deep, *n* (%) *	130 (49.8)	64 (51.7)	0.43
Infratentorial, *n* (%) *	47 (18)	9 (7.6)	0.01
IVH, *n* (%) *	170 (65.1)	99 (83.9)	0.0002
Hydrocephalus, *n* (%) *	96 (36.8)	66 (55.9)	0.001

SD: standard deviation, IQR: interquartile range, APACHE II: Acute Physiology and Chronic Health Evaluation II, PEEP: positive end-expiratory pressure, EVD: external ventricular drain, ICH: intracerebral hemorrhage, IVH: intraventricular hemorrhage. * upon admission, ** within the first 24 hours, *** during inpatient treatment.

**Table 3:** Cox Regression Hazard Model in relation to intrahospital mortality.

Parameter	Survivor (*n* = 261)	Non-Survivor (*n* = 118)	Hazard Ratio	95% CI	*p*-Value
Age, years, Mean (± SD) *	66.4 (13.5)	72.4 (11.8)	1.035	1.014–1.055	0.01
Glasgow Coma Scale Score, Median (IQR) *	10 (6–13)	4 (3–7)	0.827	0.767–0.892	<0.0001
APACHE II Score, Median (IQR) *	13 (10–15)	20 (18–22.3)	1.182	1.130–1.236	<0.0001
Heart Failure, *n* (%) *	11 (4.2)	12 (10.2)	1.558	0.791–3.07	0.2
Absence of Premedication, *n* (%) *	101 (38.7)	74 (62.7)	1.612	1.000–2.601	0.05
Antihypertensive Drugs, *n* (%) *	137 (52.5)	39 (33.1)	1.031	0.664–1.601	0.89
Norepinephrine Application Rate, µg/kg/min, Mean (± SD) **	0.02 (0.04)	0.03 (0.02)	1.033	0.975–1.094	0.27
Inspiratory Oxygen Fraction, Mean (± SD) **	0.34 (0.14)	0.37 (0.12)	0.972	0.954–0.991	0.004
Body Temperature, Centigrade, Median (IQR) *	36.4 (35.8–37)	35.9 (35.1–36.5)	1.013	0.875–1.172	0.87
Blood Glucose, mg/dl, mean (± SD) *	157.9 (54.8)	176.3 (66.6)	1.004	1.001–1.007	0.14
C-Reactive Protein/Albumin Ratio, Mean (± SD) *	0.4 (0.9)	0.9 (1.5)	1.153	1.005–1.322	0.42
ICH Volume, cm^3^, Mean (± SD) *	42.3 (34.5)	72.9 (49.9)	1.008	1.005–1.012	<0.0001
IVH, *n* (%) *	170 (65.1)	99 (83.9)	1.261	0.757–2.101	0.37

95% CI: 95% confidence interval, SD: standard deviation, IQR: interquartile range, APACHE II: Acute Physiology and Chronic Health Evaluation II, ICH: intracerebral hemorrhage, IVH: intraventricular hemorrhage. * upon admission, ** within the first 24 hours.
